# A high-density linkage map and sex-linked markers for the Amazon Tambaqui *Colossoma macropomum*

**DOI:** 10.1186/s12864-021-08037-8

**Published:** 2021-10-02

**Authors:** Eduardo Sousa Varela, Michaël Bekaert, Luciana Nakaghi Ganeco-Kirschnik, Lucas Simon Torati, Luciana Shiotsuki, Fernanda Loureiro de Almeida, Luciana Cristine Vasques Villela, Fabrício Pereira Rezende, Aurisan da Silva Barroso, Luiz Eduardo Lima de Freitas, John Bernard Taggart, Herve Migaud

**Affiliations:** 1 Embrapa Pesca e Aquicultura, Prolongamento da Av. NS 10, Cruzamento com AV. LO 18, Sentido Norte, loteamento Água Fria, CEP, Palmas, TO 77008-900 Brazil; 2 Institute of Aquaculture, Faculty of Natural Sciences, University of Stirling, Stirling, Scotland FK9 4LA UK; 3 Embrapa Amazônia Ocidental, Manaus, AM CEP 69010-970 Brazil

**Keywords:** Linkage map, Sex-linked markers, Monosex, Sex determination, *Colossoma macropomum*, Aquaculture

## Abstract

**Background:**

Tambaqui (*Colossoma macropomum*, Cuvier, 1818) is the most economically important native freshwater fish species in Brazil. It can reach a total length of over 1 m and a weight of over 40 kg. The species displays a clear sex dimorphism in growth performance, with females reaching larger sizes at harvest. In aquaculture, the production of monosex populations in selective breeding programmes has been therefore identified as a key priority.

**Results:**

In the present study, a genetic linkage map was generated by double digest restriction-site associated DNA (ddRAD) sequencing from 248 individuals sampled from two F1 families. The map was constructed using 14,805 informative SNPs and spanned 27 linkage groups. From this, the tambaqui draft genome was improved, by ordering the scaffolds into chromosomes, and sex-linked markers were identified. A total of 235 markers on linkage group 26 showed a significant association with the phenotypic sex, supporting an XX/XY sex determination system in the species. The four most informative sex-linked markers were validated on another 206 sexed individuals, demonstrating an accuracy in predicting sex ranging from 90.0 to 96.7%.

**Conclusions:**

The genetic mapping and novel sex-linked DNA markers identified and validated offer new tools for rapid progeny sexing, thus supporting the development of monosex female production in the industry while also supporting breeding programmes of the species.

**Supplementary Information:**

The online version contains supplementary material available at 10.1186/s12864-021-08037-8.

## Background

Over the past decade, the Brazilian aquaculture industry has undergone great changes, with expansion in the production of native species at a yearly growth rate of 22% mainly to supply regional markets [1, 2]. The high competitiveness of globally produced aquaculture species has driven technological innovations in the Brazilian aquaculture sector to reduce production costs, increase productivity, and diversify fish products to meet market demands [2, 3]. The tambaqui *Colossoma macropomum* (Cuvier, 1818), also known as the “black pacu”, has been identified as the most promising native species for the expansion of aquaculture in Brazil [4], with current production exceeding 139,000 t across approximately 4000 production units. The species accounts for more than 29% of fish aquaculture in Brazil [1], with an estimated increase in production of 68% by 2021 [5]. Alternatively, *C. macropomum* and its hybrids tambacu (female *C. macropomum* × male *Piaractus mesopotamicus*) and tambatinga (female *C. macropomum* × male *P. brachypomus*) are also produced in Brazilian aquaculture [1]. *C. macropomum* has been selected as the main candidate native species for genetic improvement programmes due to its many attributes for aquaculture, including a high market value, relatively easy and well controlled reproduction in captivity, excellent growth potential, good resilience to different rearing systems and an omnivorous diet [2–4].

*C. macropomum* growth and morphometric traits have great potential for selection since moderate to high heritability has been reported with genetic gains ranging from 8 to 31% [6–8]. However, the rate of genetic gain for body size is reduced in mixed sex populations due to a strong sex dimorphism in growth in favour of females. After sexual maturation, females can be 16% heavier that males of the same age [8, 9]. Importantly, differences in body weight between genders are not observed during the grow-out period (1.5–2.0 kg) when animals are usually phenotyped for selective breeding. For this reason, heritability and selection indices may be overestimated when gender is not accounted for [8].

Sex dimorphism, especially in growth rate, has been reported in many aquaculture finfish species with females usually reaching bigger sizes than males, including Atlantic halibut, *Hippoglossus hippoglossus* [10], Atlantic sea bass, *Dicentrarchus labrax* [11] and Japanese flounder, *Paralichthys olivaceus* [12] but also in crustaceans species like the giant freshwater prawn, *Macrobrachium rosenbergii* [13] and swimming crab, *Portunus trituberculatus* [14]. Therefore, the production of all-female stocks would significantly improve the productivity and profitability of these species [13, 15, 16]. Indirect hormonal manipulations are usually effective at producing all-female populations in species with a heterozygous sex determination system with females as the homogametic sex (i.e., XX/XY female/male [15];). Previous study has identified 2*n* = 54 chromosomes in tambaqui and their hybrids by cytogenetic techniques, however any heteromorphism has been observed for sex chromosomes [17]. In *C. macropomum*, the sex determination system remains unknown, but a basic protocol for monosex female production through direct feminisation using 17 β-oestradiol has been published recently [18]. However, direct sex reversal is banned in many countries across the globe due to concerns for workers on farms handling hormones, discharge into the environment and food safety for consumers [19]. Indirect sex reversal protocols can be developed if tambaqui sex determination is proven to be heterozygous and the identification and validation of sex markers would fast track progeny testing and implementation in the industry.

Linkage mapping is critical for identifying the location of regions related to quantitative traits, such as those involved in disease resistance, growth, and sex determination. Previous study has obtained a large-scale SNP discovery to build a high-density linkage map in tambaqui (2811 cM), but they have not involved quantitative traits [20]. Marker-assisted selection and genomic selection using genetic markers linked to specific quantitative trait locus (QTL) affecting a trait of commercial interest have great potential to improve selection accuracy and accelerate genetic gain through selective breeding in many aquaculture species [21–23]. Marker-assisted selection and genomic selection have already been applied successfully to several aquaculture species for traits such as sexual maturity and disease resistance [23, 24]. Sex markers can be used as a tool to identify sex during the grow-out stage within selective breeding programmes and improve selection accuracy and genetic gains.

In the present study, double digest restriction-site associated DNA (ddRAD) (ddRAD) sequencing was employed to construct a high-density genetic linkage map for association analysis of sex-linked QTL in tambaqui. Sex-linked markers were identified and validated by fluorescent based, allele specific PCR technology, to provide a tool for future development of monosex aquaculture and selective breeding programmes.

## Results

### Genome survey summary and markers assembly

High throughput sequencing of the 248 individuals from two families produced 933,251,367 raw paired-end reads in total. Reads were deposited at the EBI European Nucleotide Archive (ENA) project PRJEB33856. After removing low-quality reads and demultiplexing, 68.84% of the total reads were retained. The sequences were aligned with the *C. macropomum* genome scaffolds and genotypes for all samples were obtained using Stacks software, yielding 6,055,367 unique loci. Mean coverage per locus was 81.9x (Min: 4.4x, Max: 423.7x). Three samples (F01_143, F01_072 and F01_045) with a very low coverage and very high rate of missing sites were excluded from further analysis (Supplementary Table S1). Heterozygosity of the markers was low, as only 1.4% of the shared loci were polymorphic. A total of 19,293 polymorphic loci which were identified in the parents and at least 75% of the progeny were subsequently used to construct genetic linkage maps and perform a sex association analysis (Table 1).

**Table 1:** Sequencing summary

Category	Number/length
Total number of reads	1,866,502,734
Total number of bases	279,975,410,100 nt
Read length	150 nt
Total number of filtered reads	1,285,083,893
Unique RAD tags	6,055,367
Polymorphic SNP markers	19,293
Informative SNP markers	14,805
Genetic map (*averaged sex*): loci	5459
Average-sex size	2751.81 cM

### Linkage map construction

**Fig. 1:**
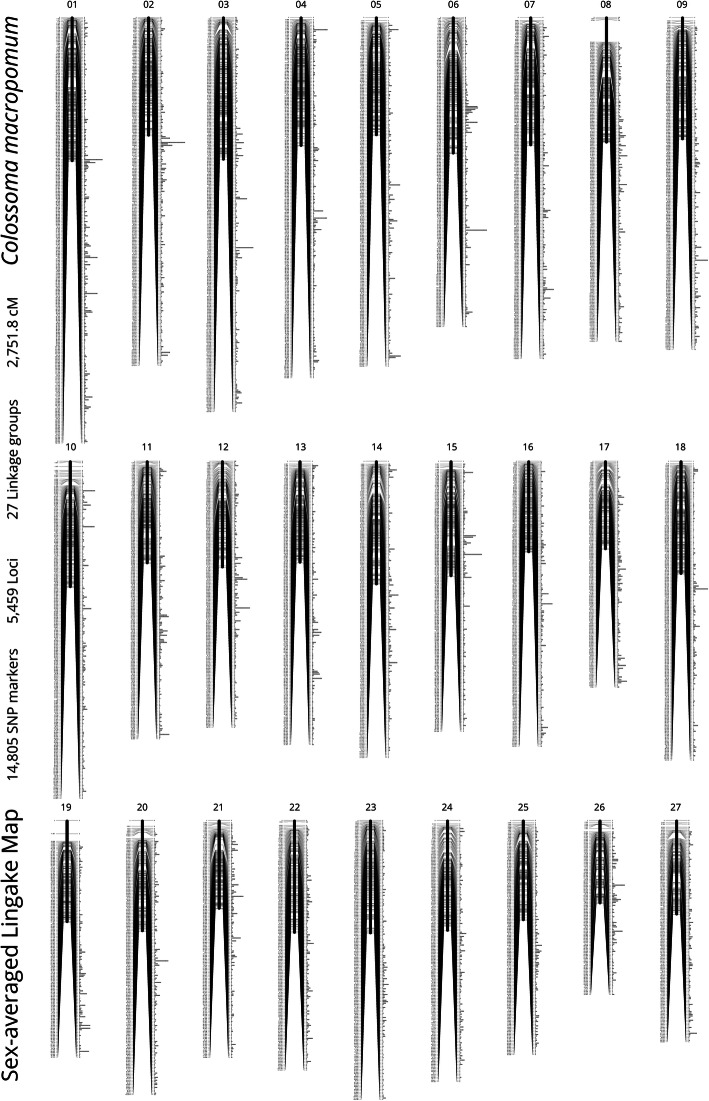
Sex-Averaged linkage map of *C. macropomum*. The 27 linkage groups are ordered by number of SNP markers. In each linkage group, numbers shown on the left provide the position (in cM) of the respective locus on the chromosome, while bars on the right indicates the relative number of SNP markers

In total, 14,805 informative SNPs were mapped to the expected 27 linkage groups, from the karyotyping [25], by using LepMap3 with a threshold logarithm of the odds (LOD) value of 11. Sex-averaged, female, and male genetic linkage maps were constructed (Fig. 1, Supplementary Fig. S1). The sex-averaged map was spanning a total distance of 2752 cM with an average inter-locus distance of 0.51 cM (Table 2). The number of markers in a linkage group varied from 395 (LG 27) to 917 (LG 1) with an average of 548, and the genetic length per group ranged from 75.09 cM (LG26) to 130.20 cM (LG 1) with an average of 101.92 cM. The female map comprised 3147 loci and spanned 2733 cM, with an average loci interval of 0.90 cM; the male map consisted of 3022 loci and spanned 2925 cM with an average loci interval of 0.98 cM (Table 2 and Supplementary Table S2). The ordering and orientation of the *C. macropomum* genome scaffolds to reconstruct chromosomes (Supplementary Data S1) were performed using the linkage maps (sex average).

**Table 2:** Summary of the genetic linkage map of *C. macropomum*. * Estimated length (gap length between or within scaffold are unknown)

Chr./LG	Chr. Length* (bp)	Markers	Average-sex Loci	Average-sex Size (cM)	Female Loci	Female Size (cM)	Male Loci	Male Size (cM)
1	67,510,651	917	297	130.20	143	133.83	180	135.22
2	52,904,932	716	243	107.07	134	125.43	122	104.51
3	52,784,634	694	275	128.79	157	124.36	155	134.98
4	45,648,328	638	251	116.36	147	94.80	119	114.29
5	45,481,260	641	242	106.74	149	108.94	117	109.39
6	47,130,658	623	216	123.25	119	124.65	112	130.37
7	40,559,550	598	237	115.84	145	106.67	117	129.57
8	46,777,390	598	212	113.34	109	115.85	125	110.87
9	49,677,613	595	230	110.42	156	96.87	106	120.64
10	44,348,188	581	231	113.92	147	91.91	107	125.02
11	37,564,635	561	194	92.38	108	94.58	110	95.72
12	38,025,399	543	186	96.11	100	89.39	105	98.83
13	40,757,001	545	197	91.79	123	100.14	102	96.78
14	36,781,461	524	206	111.42	134	102.63	97	109.51
15	42,853,313	522	187	104.01	112	97.55	106	105.00
16	44,064,100	498	199	82.33	119	82.75	101	109.89
17	35,989,072	492	158	79.64	92	69.70	89	90.24
18	43,836,131	488	208	101.97	119	145.40	132	178.93
19	40,668,426	477	153	92.04	102	76.16	93	90.28
20	40,163,545	479	187	100.24	106	116.80	115	86.69
21	41,776,805	481	165	79.90	78	84.78	99	78.51
22	34,436,174	462	173	101.63	92	99.16	117	105.80
23	34,642,816	454	195	102.18	120	104.55	116	97.60
24	33,779,548	450	181	99.80	105	92.91	104	96.14
25	36,164,054	418	163	90.29	100	84.89	86	95.91
26	31,836,560	415	120	75.09	67	74.20	70	104.30
27	33,389,210	395	153	85.09	64	94.33	120	70.20
Unplaced	82,308,352	–	–	–	–	–	–	–
Total	1,221,859,806	14,805	5459	2751.81	3147	2733.20	3022	2925.19

### Association analysis and QTL mapping

**Fig. 2:**
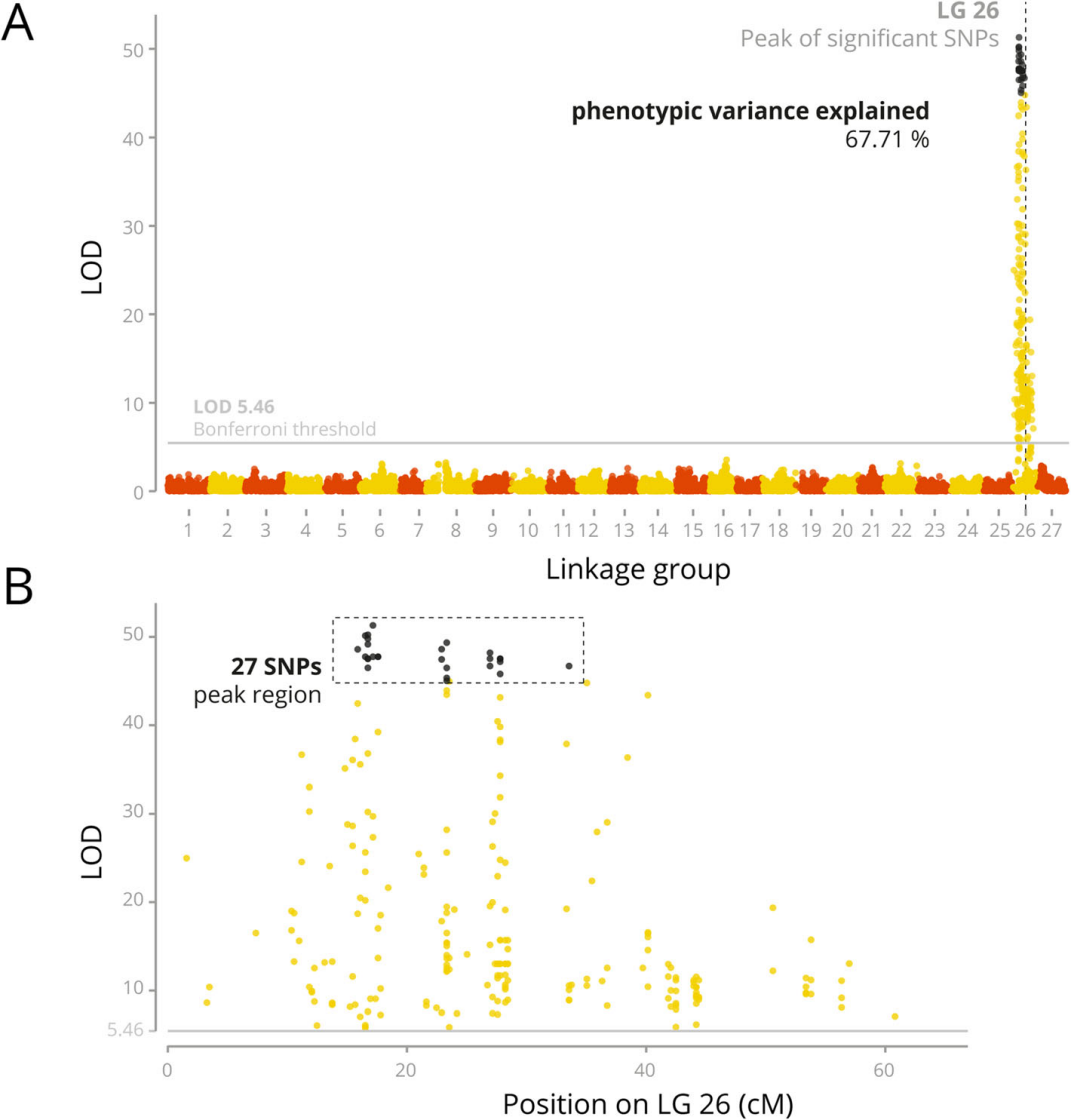
Genome wide association results for genotyped markers. The LOD score for association of directly genotyped SNPs are plotted as a function of position of the genetic map. Each linkage group (LG) has been represented with different colour SNPs, with *p*-values achieving genome-wide significance. The sex-linked markers with the highest LOD scores (> 45) are shown in orange. A) Full Manhattan plot; B) Details of the significant makers on LG 26

R/SNPassoc software was used to conduct a quantitative trait locus (QTL) mapping analysis for sex determination association. QTL fine mapping based on high-density genetic linkage maps provided evidence for the existence of a major QTL in LG 26 for both families. The result for genome-wide significant QTL was identified on LG 26 (Fig. 2). From the 415 markers on LG 26, a total of 239 (57.6%) were strongly associated with sex with *P* < 10^− 6^. After Bonferroni correction for multiple tests, the significant LOD score threshold was 5.46. The highest LOD values (over 45) from 27 markers were observed in a region ranging from 15.9 cM (LOD = 48.6, scaffold NW_023494809.1:1051596) and 33.6 cM (LOD = 46.7, scaffold NW_023494809.1:15347236), representing an interval of 17.7 cM or 14,295,640 bp (based on alignment of the markers on the final genome).

### Verification of SNP sex-association and validation

Primers for PACE assays were designed for the four potentially most associated sex-linked markers (Table 3 and Supplementary Table S3). All four markers were assessed in extra samples collected for this validation step (22 broodstock and their 184 F1 progeny) using PACE SNP genotyping (Supplementary Table S4). The markers Cma511969, Cma5145911, Cma5119452 and Cma5117879 showed between 90.0 and 96.7% association to the phenotypic sex (Table 4).

**Table 3:** Selected SNP markers. The four markers significantly associated with phenotypic sex based on LOD score and position on LG 26

Marker	Chr./LG	Chr. position	Position Average	Position Female	Position Male	LOD	Male	Female
Cma5119693	26	1,051,596	15.88	58.77	49.96	48.59	C/G	G/G
Cma5119452	26	1,002,040	16.73	58.77	51.24	49.79	A/G	A/A
Cma5117879	26	4,967,169	16.51	60.90	59.32	50.13	A/G	G/G
Cma5145911	26	6,424,206	16.51	58.77	49.96	47.75	C/T	C/C

**Table 4:** Validation data of sex markers in *C. macropomum* broodstock and their offspring archived by PACE and ddRAD showing accuracy

Genotyping Approach	Sample type	Marker	Accuracy (%)
ddRAD assay	Offspring	Cma5145911	96.06
ddRAD assay	Offspring	Cma5119693	97.30
ddRAD assay	Offspring	Cma5119452	95.74
ddRAD assay	Offspring	Cma5117879	94.22
PACE assay	Broodstock	Cma5145911	90.00
PACE assay	Broodstock	Cma5119693	90.00
PACE assay	Broodstock	Cma5119452	90.00
PACE assay	Broodstock	Cma5117879	90.00
PACE assay	Offspring	Cma5145911	96.15
PACE assay	Offspring	Cma5119693	96.17
PACE assay	Offspring	Cma5119452	96.72
PACE assay	Offspring	Cma5117879	96.71

## Discussion

In this study, we identified 14,805 informative SNPs and constructed linkage maps for *C. macropomum*, which represents the highest density genetic map so far generated for this species. The approach enabled to map sex-associated region on a single chromosome (LG 26) supporting an XX/XY sex determination system in tambaqui. A panel of four sex related markers was successfully validated in a wider population for use in future selection and monosexing within breeding programme in the species.

The construction of high-density, informative genetic linkage maps is an essential pre-requisite for resolving QTLs associated to traits of interest in aquaculture species. In recent years simultaneous discovery and genotyping of SNP loci by RAD-based technologies has been widely exploited for linkage and QTL mapping due to its relative simplicity (not requiring any prior genomic reference), multiplexing capacity, flexibility and cost efficiency [26]. One variant, ddRAD, has been deployed successfully across a range of aquaculture species to study population structure, fish sex determination and identify sex linked genetic markers for sex genotyping assays [10, 14, 27–30]. In the present study, we developed a high-density genetic linkage map by ddRAD sequencing in tambaqui, identified a strong sex associated QTL in LG 26, provided data to support an XX/XY female/male heterozygous sex determination system and validated a panel of SNPs to assign sex in future studies.

Three genetic linkage maps were constructed for tambaqui covering a total distance of 2752 cM, 2733 cM and 2925 cM for sex-averaged, female and male maps, respectively. No significant differences were observed between maps. The total number of markers in the consensus map was 14,805 SNPs, covering 5459 loci, and the average loci interval of the sex-averaged map was 0.51 cM. These results agree with a previous linkage map published for tambaqui based on a genotyping-by-sequencing approach made without accounting for sex (2811 cM of total distance coverage, 7192 mapped SNPs with a 0.39 cM interval) and without QTL mapping analyses [20]. Heterozygosity recorded was only of 1.4%. However, this is likely not representative of the species, but of breeding lines used in aquaculture. Before fish rearing in Embrapa, the founder populations originated from commercial captive broodstocks bred over several generations and therefore explaining the current population genetic polymorphism observed. This is an important consideration for future breeding programmes in the species as low heterozygosity may be a result of inbreeding.

In the current study, one major QTL (LG 26) related to sex determination was supported by four markers (LOD > 45) that were found to peak in one single region (15.88 cM and 16.51 cM) of LG 26. Together, they contribute to 67.71% of the phenotypic variation, suggesting the existence of a sex-linked QTL in tambaqui. Further genotyping of the sex-linked QTLs revealed that male genotypes were heterozygous, whereas females were homozygous. Validation of the four successfully amplified sex associated markers in broodstock (*n* = 22) and their progeny (*n* = 184) revealed an accuracy ranging from 90.0 to 96.7% at correctly predicting sex. Inconsistencies in sex assignment from SNP assays of sex-linked markers between different sires and dams have been reported in other species including Atlantic halibut [10], Nile tilapia, *Oreochromis niloticus* L. [27] and swimming crab [14]. This may be partly explained by the lack of recombination suppression in regions that include and flank the sex determining region in these species, so that the detected sex associated markers may frequently cross over during meiosis. Nevertheless, the sex-associated SNPs provide clear evidence that those markers are in strong linkage disequilibrium with the sex-determining genes and suggest that tambaqui has an XX/XY sex determination system.

The morphology of tambaqui chromosomes is metacentric and submetacentric; nevertheless, no heteromorphism in sex chromosomes has been observed [17]. In fish, sex determining QTLs are often found in sub-telomeric regions, suggesting that chromosome ends are areas of accelerated evolution developing non-recombining via rearrangements [31]. As a result of recombination suppression, these chromosomes are derived from autosomal regions of the genomes with higher plasticity and lower density of core genes, and are rich in transposable elements and other repetitive sequences [31, 32]. Our results are consistent with the above with blocks of suppression found at around 11 cM of distance on the LG 26, without enzymes restriction sites used by ddRAD, however, this region is not large enough to indicate sexual chromosome heteromorphism.

## Conclusions

The PACE-validated sex marker panel used in this study is a powerful tool for gender monitoring and increasing genetic gains in future selective breeding programmes of tambaqui. As a result, the sex-linked markers identified in this study provide a valuable molecular tool for discriminating genders early in the production cycle. Furthermore, sex markers will expedite the identification of neomales following indirect hormonal sex reversal, which appears to be achievable given the species’ apparent XX/XY sex determination mechanism.

## Methods

### Family construction and rearing conditions

Five full-sib *C. macropomum* families were reared at the facilities of Embrapa Fisheries and Aquaculture, Palmas-TO, Brazil. The families, FAM01, AM01, EMB01, EMB02 and EMB04, were reared in separate 1 m^3^ tanks for 175 and 215 days, respectively. Fish were fed twice a day ad libitum with a commercial dry pellet diet containing 32% crude protein during the first 60 days, and 28% crude protein thereafter (Guabi S. A, Brazil).

### Sample collection for QTL mapping and sex-linked SNP markers validation

At sampling, a total of 244 F1 juveniles from two families (142 from FAM01 and 102 from EMB01) were randomly culled by lethal anaesthesia (10% Benzocaine solution; Merck & Co., USA), and a fin clip was collected, fixed in 100% ethanol, and kept at − 20 °C until DNA extraction. Fish were then dissected and gonad samples for histological identification of sex were fixed in Bouin’s solution for 24 h, then washed in distilled water and dehydrated in a series of ethanol solutions (70, 80, 90 and 100%) according to Almeida et al. [9]. Gonad samples were embedded in paraffin wax, sectioned (5 μm), mounted on slides, stained with haematoxylin-eosin, and analysed under a light microscope (Leica DM500, Heerbrugg, Switzerland).

For sex-QTL validation, fin clips from another 206 individuals were further collected and fixed in 100% ethanol. It included, 22 adult broodstocks (11 males and 11 females) from commercial broodstocks from Mato Grosso State (Brazil) and maintained in Embrapa’s Germplasm Active Bank-BAG (Palmas-TO, Brazil), which were sampled after anaesthesia (10% Benzocaine solution; Merck & Co., USA). As well as an extra 184 F1 individuals, belonging to the five full-sib families (AM01 (*n* = 69), EMB01 (*n* = 20), EMB02 (*n* = 39), EMB04 (*n* = 36), FAM01 (*n =* 20)) reared in a communal tank after being tagged intramuscularly with PIT tags (Animal Tag, Brazil). Fish were then sacrificed (378 days post hatch) to dissect their gonad and identify sex histologically, as described above.

### Genomic DNA extraction

All fin clips samples were stored at 4 °C in 100% ethanol prior to use. Total DNA was extracted by a modified salt-based extraction protocol according to A Blanquer [33], as described by Taslima et al. [34]. Each sample was initially quantified by spectrophotometry (Nanodrop 1000, Thermo Fisher Scientific, USA). Genomic DNAs were and stored at 4 °C. Agarose gel electrophoresis (0.8%) was used to check the integrity of the genomic DNA. For those samples used for library construction, the DNAs were re-quantified by fluorimetry, (Qubit 2.0, Thermo Fisher Scientific, USA) and diluted further, to 7 ng/μL, in 5 mM Tris, pH 8.0.

### ddRAD library preparation and sequencing

Two ddRAD libraries were constructed, one for each family pedigree. DNAs from 142 F1 progeny and both parents were used for the FAM01 library and another 102 F1 progeny and both parents were used for EMB01 library. The methodology followed the original protocol reported by Peterson et al. [35], with some modifications/refinements as described in full by Manousaki et al. [36]. Each progeny sample was processed in duplicate while five samples from each parent were processed. Briefly, using 96 well microplates, each DNA sample (15 ng DNA per sample) was simultaneously digested by two high fidelity restriction enzymes: *Sbf*I (CCTGCA|GG recognition site), and *NIa*III (CATG| recognition site), both sourced from New England Biolabs (NEB, UK). Digestions were incubated at 37 °C for 60 min using 0.3 U of each enzyme per sample (20 U/μg DNA equivalent) in 1× CutSmart Buffer (NEB, UK), in a final reaction volume of 6 μL. A heat inactivation step was then performed at 65 °C for 25 min. After cooling the reactions to room temperature, 3 μL of a premade, unique barcode / adapter mix was added to each digested DNA sample, and incubated at 22 °C for 10 min. This adapter mix comprised individual-specific barcoded combinations of P1 (*Sbf*I-compatible) and P2 (*Nla*III-compatible) adapters at 6 nM and 1.54 μM concentrations respectively, in 1x reaction buffer 2 (NEB, UK). Adapters were compatible with Illumina sequencing chemistry [35]. The adapters included an inline five- or seven-base barcode for sample identification (Supplementary Table S1). Following a 10-min incubation, 3 μL of ligation mix was then added, comprising 4 mM rATP (Promega, UK), and 3000 cohesive-end units of T4 ligase (NEB, UK) in 1x CutSmart buffer. An optimised three steps ligation incubation was performed, i.e., 1.5 h at 14 °C, 1.5 h at 22 °C, and then overnight (16 h) at 5 °C. To stop ligation, 35 μL P1 buffer (Qiagen, UK) was added to each reaction well, the samples then being combined into a single pool. This pooled sample was column purified (MinElute PCR Purification Kit, Qiagen, UK), being eluted in 55 μL EB buffer (Qiagen, UK). Size selection of fragments was performed by agarose gel separation and excision of the 400 to 700 bp range. Following gel purification, the eluted size-selected template DNA was amplified by PCR (13 cycles). The PCR reactions were column-purified in MinElute PCR Purification Kit (Qiagen, UK). The 55 μL eluted in EB buffer was then subjected to an additional clean-up / concentration using AMPure XP magnetic beads (Beckman Coulter), being eluted in 17 μL EB buffer, quantified by fluorimetry and size checked by agarose gel electrophoresis. The libraries were commercially sequenced (Novagene, UK) on an Illumina HiSeq 2500 platform using 150 base paired-end reads (v3 chemistry).

### Genotyping ddRAD alleles

The sequences were pre-processed to discard low quality (i.e., with a quality score of less than 20), missing tag structure or ambiguous bases. Remaining reads were aligned to *C. macropomum* genome scaffolds (Assembly GCA_904425465.1) using bwa v0.7.17 [37], then sorted into loci and genotypes using Stacks v2.41 [38]. Informative markers were kept only when presenting at least two alleles with a minor allele frequency over 0.01 and which were present in both parents and at least 75% of the offspring to minimise the amount of missing or erroneous data. Only one SNP (selected at random) was reported for each RAD locus.

### Construction of linkage maps

Based on the SNP genotypes obtained, sex-specific and sex-averaged linkage maps were constructed with LepMap3 [39]. SNPs deviating from expected Mendelian segregation (*P* < 0.001) were excluded. Based on available karyotyping data from Nakayama et al. [25], the number of linkage groups (LG) was set to 27. The total length of maps in centi-Morgans (cM) was estimated using the Kosambi mapping function [40]. Maps generated with the OrderMarker2 module were checked for contiguous sequence (contig) continuity. Genetic maps were drawn using Genetic-Mapper v0.13 [41].

### Genome scaffold ordering

The ordering and orientation of the *C. macropomum* genome scaffolds to reconstruct chromosomes were performed with ALLMAPS [42] using the linkage maps (sex average).

### Association analysis and QTL mapping

Using phenotypic sex data, an association analysis was performed within the package R/SNPassoc v1.9–2 according to González et al. [43] to test for associations between SNP genotypes and phenotypic sex under a binary codominant genetic model, using the function *WGassociation*. Bonferroni correction was used to counteract the problem of multiple comparisons when determining the significance of observed results. The phenotypic variance explained (PVE) was defined as:

$$ PVE=100\times \left(1-{10}^{\frac{-2\times LOD}{N}}\right) $$

With N the number of sexed individual (*N* = 206) and LOD the peak LOD value.

### Verification of SNP sex association by PACE assay

Marker sex association was assessed for four SNPs that were commonly found in the mapping families to span the region of the highest association with sex from ddRAD-seq, using fluorescent, allele specific, endpoint-genotyping assays: PCR Allele Competitive Extension (PACE™ genotyping assays, 3CR Bioscience, UK). SNP-specific primer sets were designed by 3CR Bioscience. DNA was extracted from fin clips from the 206 individuals (22 broodstock and 184 offspring) using a HOTSHOT protocol adapted from Truett et al. [44]. Each genotyping assay was run in 10 μL volume containing approximately 15 ng of target genomic DNA incorporated with a PACE master mix reaction. All assays were run with the same touchdown thermal cycling programme using a Biometra TGradient thermal cycler (Biometra GmbH, Goettingen, Germany) as follows: 94 °C for 15 min followed by 10 cycles of 94 °C for 20 s melt, 61–57 °C for 1 min anneal and extension (decreasing of 0.6 °C per cycle) followed by 25 cycles of 94 °C for 20 s melt, 57 °C for 1 min anneal and extension. Thereafter, assays results were read at 25 °C using an endpoint genotyping programme on a Techne Quantica qPCR thermal cycler (Bibby Scientific Ltd., Stone, UK) in which unknown genotypes were assigned based on fluorescent output in comparison to non-template control wells containing DNA/RNA free H_2_O.

## Supplementary Information

**Additional file 1: Supplementary Figure S1** Linkage map of *C. macropomum*. The 27 linkage groups are ordered by number of SNP markers. In each linkage group, numbers shown on the left provide the position (in cM) of the respective locus on the chromosome, while bars on the right indicates the relative number of SNP markers. A) Female-only linkage map; B) Male-only linkage map.

**Additional file 2: Supplementary Table S1** Origin of samples and barcode. Details of each sample used: barcode, file name ID, family, gender, status number of extracted and retained reads.

**Additional file 3: Supplementary Table S2** Genetic maps. Ordered markers: marker ID, linkage group and Position Average (cM), Scaffold, Scaffold position, and Marker sequence.

**Additional file 4: Supplementary Table S3** Primer used in PACE validation.

**Additional file 5: Supplementary Table S4** Details of the PACE assay results. Genotypes of 206 samples.

**Additional file 6: Supplementary Data S1** Structure of chromosome-level genome assemblies ordered from the linkage map. AGP (A Golden Path) file formatted, reporting the scaffold structure of each linkage group into 27 chromosomes.

## Data Availability

The datasets generated and analysed during the current study are available in the supplementary information files of the manuscript.

## Abbreviations

LG: Linkage groups
cM: Centi-Morgan
LOD: Logarithm of the odds
PACE: PCR Allele Competitive Extension
ddRAD: Double digest restriction-site associated DNA
QTL: Quantitative trait locus
