# Supplementary figures and images for: A high-density linkage map and sex-linked markers for the Amazon Tambaqui Colossoma macropomum
Source: BMC Genomics. 2021 Oct 2;22:709. doi: 10.1186/s12864-021-08037-8 (PMC8487117; doi:10.1186/s12864-021-08037-8)

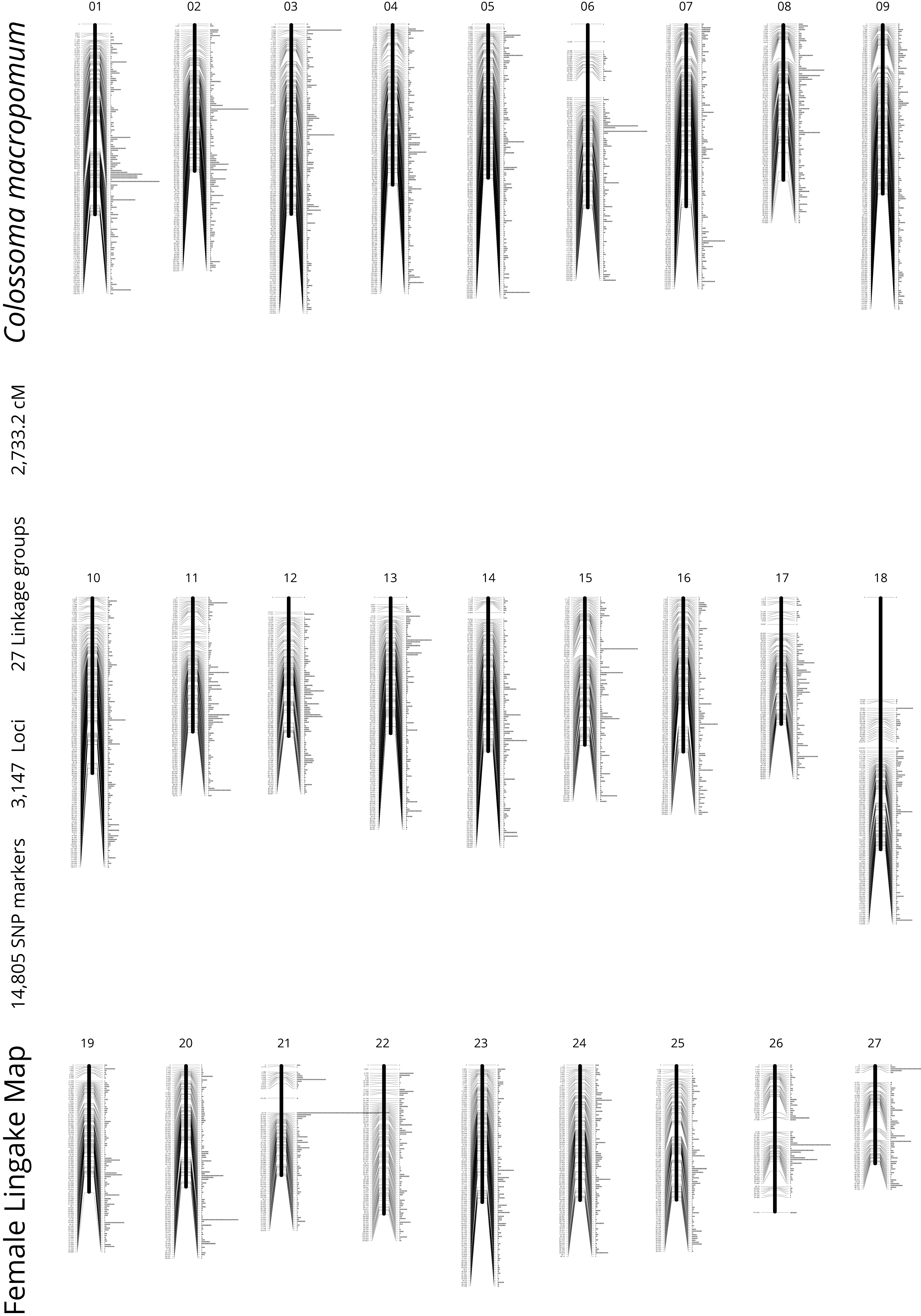

Supplement: Supplementary file 1 — Additional file 1: Supplementary Figure S1 Linkage map of C. macropomum. The 27 linkage groups are ordered by number of SNP markers. In each linkage group, numbers shown on the left provide the position (in cM) of the respective locus on the chromosome, while bars on the right indicates the relative number of SNP markers. A) Female-only linkage map; B) Male-only linkage map. [file 12864_2021_8037_MOESM1_ESM.zip › FigureS1A.v2.tif]

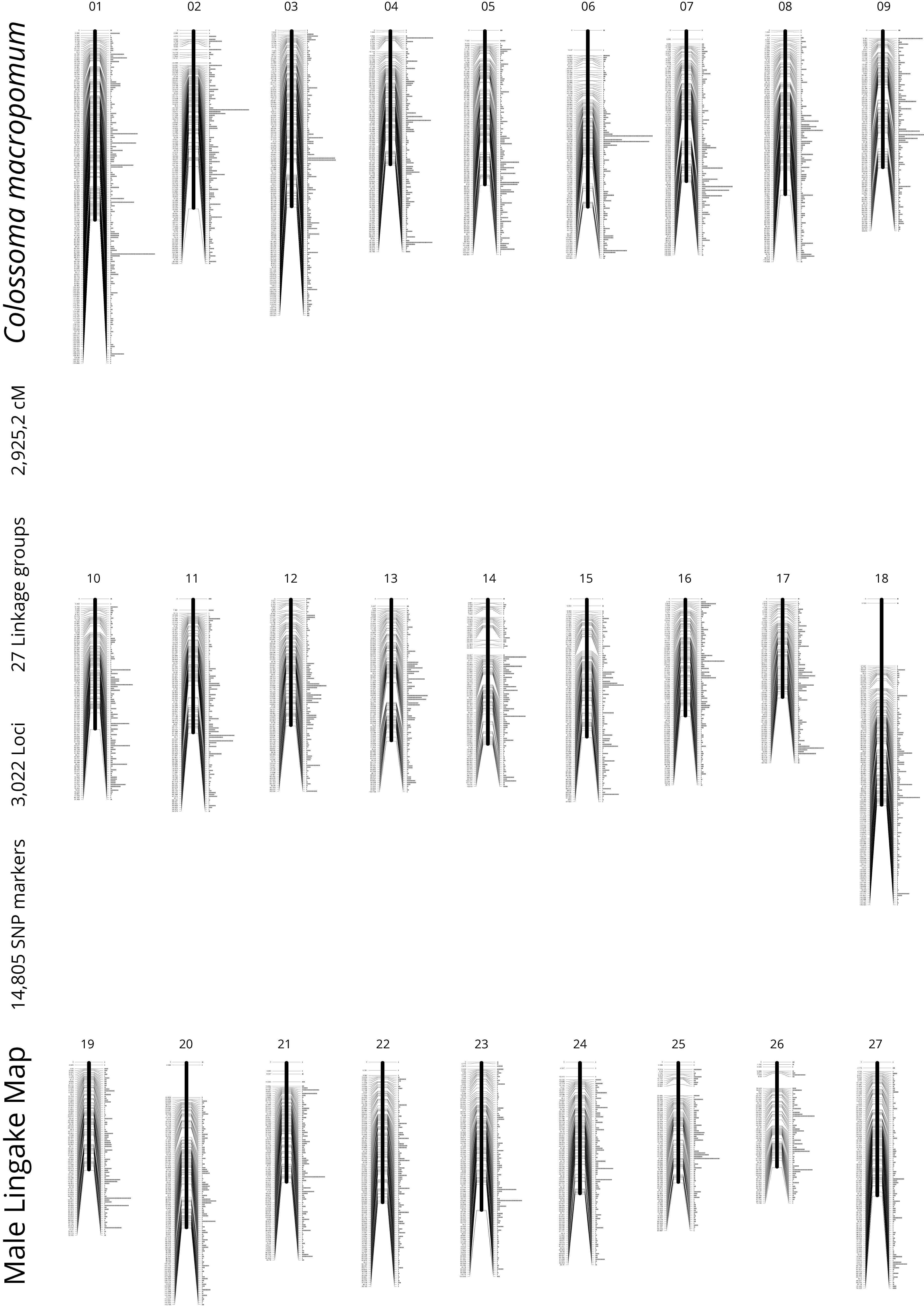

Supplement: Supplementary file 1 — Additional file 1: Supplementary Figure S1 Linkage map of C. macropomum. The 27 linkage groups are ordered by number of SNP markers. In each linkage group, numbers shown on the left provide the position (in cM) of the respective locus on the chromosome, while bars on the right indicates the relative number of SNP markers. A) Female-only linkage map; B) Male-only linkage map. [file 12864_2021_8037_MOESM1_ESM.zip › FigureS1B.v2.tif]
